# Distribution and Influencing Factors of Airborne Bacteria in Public Facilities Used by Pollution-Sensitive Population: A Meta-Analysis

**DOI:** 10.3390/ijerph16091483

**Published:** 2019-04-26

**Authors:** Eun-Min Cho, Hyong Jin Hong, Si Hyun Park, Dan Ki Yoon, Sun Ju Nam Goung, Cheol Min Lee

**Affiliations:** 1 Department of Applied Chemistry, College of Applied Science, Kyung Hee University, Yongin 17104, Korea; choeunmin@khu.ac.kr; 2 Institute of Risk Assessment, Seokyeong University, Seoul 02713, Korea; hongdonn01@skuniv.ac.kr (H.J.H.); yokkkk@naver.com (S.H.P.); ydk0207@skuniv.ac.kr (D.K.Y.); happying2@hanmail.net (S.J.N.G.); 3 Department of Nano and Biological Engineering, Seokyeong University, Seoul 02713, Korea

**Keywords:** allergen, bio-aerosol, immunodeficiency, indoor air quality, sick building syndrome

## Abstract

The aim of this study was to support management of airborne bacteria in facilities used by pollution-sensitive individuals (in daycares, medical facilities, elder care facilities, and postnatal care centers). A field survey was conducted on 11 facilities from October 2017 to April 2018. Elder care facilities in industrial, urban, and forested areas were excluded. Two indoor, and one outdoor, measuring points were selected per facility. These points were located in areas most often used by the residents. Measurements were taken at random time-points before February 2018 and at specific times in the morning and afternoon thereafter. The relationships among bacterial counts, carbon dioxide concentrations, dust levels, temperature, relative humidity, and ventilation were examined. The pooled average bacterial counts at the daycares, medical facilities, elder care facilities, and postnatal care centers were 540.25 CFU m^−3^, 245.49 CFU m^−3^, 149.63 CFU m^−3^, and 169.65 CFU m^−3^, respectively. Considering the upper 95% confidence interval, the bacterial counts in many daycares may in fact be >800 CFU m^−3^, which is the threshold set by the Korean Ministry of the Environment. The pooled average indoor: outdoor bacterial count ratio was 1.13. Indoor airborne bacterial counts were influenced mainly by their sources. This study found no significant correlations among indoor temperature, relative humidity, carbon dioxide concentration, dust levels, and airborne bacterial counts, unlike previous studies. Airborne bacteria management at daycares should be a top priority. The sources of airborne bacteria must also be identified, and a management plan must be developed to control them.

## 1. Introduction

After the global energy crises of the 1970s, building designs and constructions were regulated to meet energy efficiency criteria [1]. These regulations were economically effective in that they conserved energy, but they also reduced indoor air circulation. Consequently, the amount of harmful substances remaining indoors increased and indoor air quality declined. Studies have shown that numerous harmful pollutants have increased in buildings because of poor indoor air quality. Continuous exposure to these substances may have adverse health effects [2,3,4].

In recent years, there has been a substantial increase in the number of people complaining of the symptoms of sick building syndrome, including headache, dizziness, nausea, drowsiness, eye irritation, and the inability to concentrate. Some of these effects are caused by increases in the levels of bio-aerosols. Those who are sensitive to indoor air pollutants may develop respiratory diseases like rhinitis, asthma, and pneumonia, or inflammatory diseases with allergic symptoms, such as atopic dermatitis. In severe cases, death may occur [5,6]. Bio-aerosol exposure may exacerbate the symptoms of sick building syndrome [7,8]. Bio-aerosols include microbial pathogens (bacteria, fungi, and viruses), pollen, insects, microbial metabolites, and plant and animal debris [9,10,11]. Bio-aerosols may be ≤200 μm in diameter, remain airborne, and enter the human body by inhalation [12]. Bio-aerosols of 5–10 μm in diameter deposit in the upper respiratory tract and induce rhinitis. Bio-aerosols <5 μm in diameter traverse the alveoli and trigger allergic reactions [13]. The effects of bio-aerosols on the human body differ among individuals. These aerosols tend to be more harmful to people with weakened immunity, such as the elderly, children, and pregnant women [14].

The environmental guideline for bio-aerosols in residential and working environments were proposed by several researchers and/or recommended by various levels of government [15,16]. Nevertheless, no nation has legally regulated bio-aerosols to date. The Republic of Korea manages facilities used by groups with fragile immunity such as the elderly, children, and pregnant women, because they are at a relatively higher risk of developing adverse health effects upon exposure to bio-aerosols. Therefore, the government set a threshold of 800 CFU m^−3^ for indoor airborne bacteria. As of 2018, the government of Korea, recognizing the significance of clean indoor air, has imposed an additional limit of 500 CFU m^−3^ for indoor airborne fungi. To date, however, no systematic management measures have been undertaken to reduce airborne bacteria in domestic indoor environments. Consequently, the government of Korea has applied the control measures prevalent abroad to manage domestic indoor airborne bacteria. One aim of this study was to identify the factors influencing the distribution and concentration of airborne bacteria in facilities used by people with weakened immunity. Another objective was to help establish scientific measures to reduce exposure to systematic airborne bacteria and bio-aerosols in domestic environments.

## 2. Materials and Methods

### 2.1. Literature Review and Analysis

This study consisted of a literature review and a field survey. In the first step, literature on airborne bacterial distribution in target Korean facilities since 1991 was investigated. Factors increasing or decreasing indoor airborne bacteria levels in target facilities were evaluated by determining the relative influence of each factor and assessing the relationships among them. All statistical analyses were performed using the statistical software package, SPSS v18 (SPSS Inc., Chicago, IL, USA). Associations between airborne bacteria and influencing factors were examined by assigning the level of each influencing factor to a quartile. Differences in airborne bacterial concentrations in each category were compared with one-way ANOVA at a significance level of 0.05.

### 2.2. Facility Studied in this Study and Airborne Bacterial Sampling

Next, the concentrations of airborne bacteria in the target facilities were measured. Due to budget and time limitations, this work investigated 52 day-care centers, 49 medical centers, 35 elderly care centers, and 54 postnatal care centers in Korea. The field survey and the air sampling in each facility occurred within the same visitation period. The locations included daycares, medical facilities, elder care facilities, and postnatal care centers in industrial, urban, and forested areas. No elder care facilities were situated in industrial areas. Eleven facilities, including three daycares, three medical facilities, two elder care facilities, and three postnatal care centers were examined.

The survey was conducted once monthly in all facilities except in the daycares from October 2017 to April 2018. The daycares were surveyed once monthly from November 2017 to April 2018. The field survey was conducted at various times of the day before February 2018. To determine whether indoor airborne bacterial concentrations changed within the same day, the field survey was conducted at specific times in the morning and afternoon from February 2018 onwards. Two indoor points and one outdoor site most frequently used by the residents were selected and the surveys were run at the same time each visit. Airborne bacteria, temperature, relative humidity, fine particulate matter (PM_10_, PM_2.5_), and CO_2_ were measured.

A MAS-100 (Merck GmBH, Darmstadt, Germany) was used for airborne bacteria sampling. The sampler was mounted 1.2–1.5 m above floor level. The sampling was performed three times for 1 min every 20 min. The average was used as the representative measurement point. Before sampling, the inside of the collector was sterilized with 70% v/v alcohol and packed with sterile trypticase soy agar (Lot No. 2087730, Becton, Dickinson and Company, Franklin Lakes, NJ, USA). Once sample collection was complete, the medium was aseptically removed from the counter, sealed with Parafilm M (Sigma-Aldrich Corp., St. Louis, MO, USA), stored in an icebox, and transferred to the laboratory. The medium was incubated at 25 ± 1 °C for 48 h, and the bacterial colonies on it were counted. The colonies were calibrated using a colony count conversion table. The calibrated number of colonies was divided by the volume of air sampled to obtain the airborne bacterial concentration (CFU m^−3^).

Temperature and relative humidity were simultaneously measured using a portable thermal environment meter (TM-181; Tenmars Electronics Co. Ltd., Taipei, Taiwan). The instrument was stabilized and the values it generated were taken to represent the temperature and relative humidity of the measurement point. Fine particulate matter was measured with a direct-reading light-scattering meter, which simultaneously records PM_10_ and PM_2.5_ (AEROCET 831, Met One Instruments, Shelburne, VT, USA). Measurements were taken every minute for 30 min and the averages were taken to represent the PM_10_ and PM_2.5_ at the sampling point. CO_2_ concentration was measured with a direct-reading non-dispersive Insulation resistance (IR) meter (Geotech G150, Geotech, Coventry, UK). Measurements were taken every minute for 30 min and the averages were taken to represent the CO_2_ concentration at the sampling point.

### 2.3. Meta-Analysis

To compensate for the relative lack of reliable, representative data in the present study, a meta-analysis was conducted by linking the collected data analysis and field survey results.

**Figure 1:**
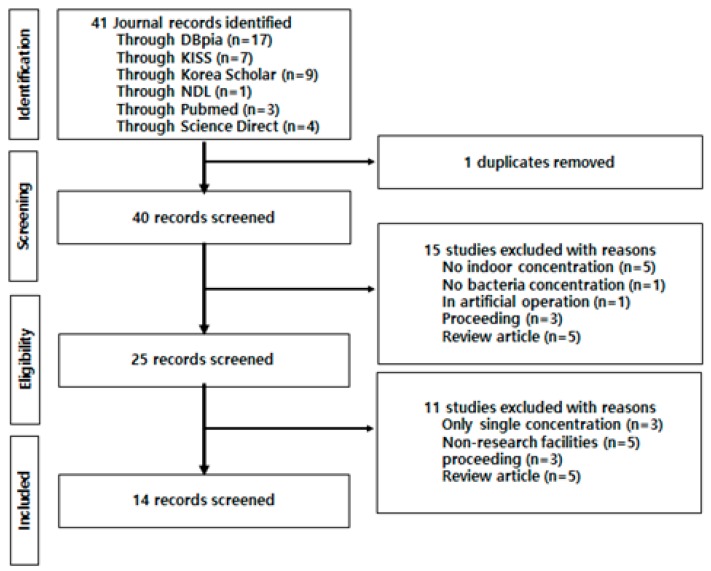
Mimetic diagram for qualitative meta-analysis using literature selection and survey.

Figure 1 shows the process of selecting data from previous studies for the meta-analysis. The findings from 25 studies on airborne microorganisms in the domestic indoor environment were used in the qualitative meta-analysis. Three studies reported only single concentrations, eight studies presented geometric means, and five studies evaluated target facilities different from those sampled in the present study. All 16 of these were excluded from the meta-analysis. Data from the remaining six studies were used in the quantitative meta-analysis.

The quantitative meta-analysis used inverse-variance-weighted average concentrations, which constitute part of an effect-magnitude measure. This method calculates the merged average by considering the scale of each study, the volume of data, and the average concentrations derived from the selected studies. This method assumes that the estimates of the merged means are approximately normally distributed. The functional formula used in this technique is as follows:

(1) $$\bar{\theta_{i}}=N\left(\theta_{i},\;\omega_{i}^{-1}\right),\;i=1,2,\ldots ,\;k$$

where $\omega_{i}$ is an inverse variance of $\bar{\theta_{i}}$, and k is independent. When the estimates of the merged means are $\theta_{1}$ = $\theta_{2}$ = … = $\theta_{k}$ = θ, the distribution follows an asymptotic normality:

(2) $$\sum^{\;}\bar{\theta_{i}}\omega_{i}\sim N\left(\theta \sum^{\;}\omega_{i},\sum^{\;}\omega_{i}\right).$$

Therefore, the merged mean and its variance are expressed as follows:

(3) $$\bar{\theta }=\frac{\sum_{i=1}^{k}\bar{\theta_{i}}\omega_{i}}{\sum_{i=1}^{k}\omega_{i}}$$

(4) $$\mathrm{var}\left(\bar{\theta }\right)=\frac{1}{\sum_{i=1}^{k}\omega_{i}}$$

where $\bar{\theta_{i}}$ follows an asymptotic normality. The 95% confidence interval of $\bar{\theta_{i}}$ was calculated using the following equation:

(5) $$\bar{\theta }\pm 1.96\sqrt{\frac{1}{\sum_{i=1}^{k}\omega_{i}}}$$

## 3. Results and Discussion

### 3.1. Concentration Characteristics of Airborne Bacteria by Facility Type

The concentration distribution of airborne bacteria was significantly higher in the daycares than in the other facilities. A post-hoc analysis showed that daycares showed significant differences in concentration distribution with those of elder care facilities and postnatal care centers but not with those of medical facilities (Table 1). Sneezing, coughing, and talking causes pollutants on indoor surfaces to float, thereby increasing the concentration of airborne bacteria [16,17,18]. The number of residents and the level of activities, like walking and running, also increase the concentration of airborne bacteria [19,20].

**Figure 2:**
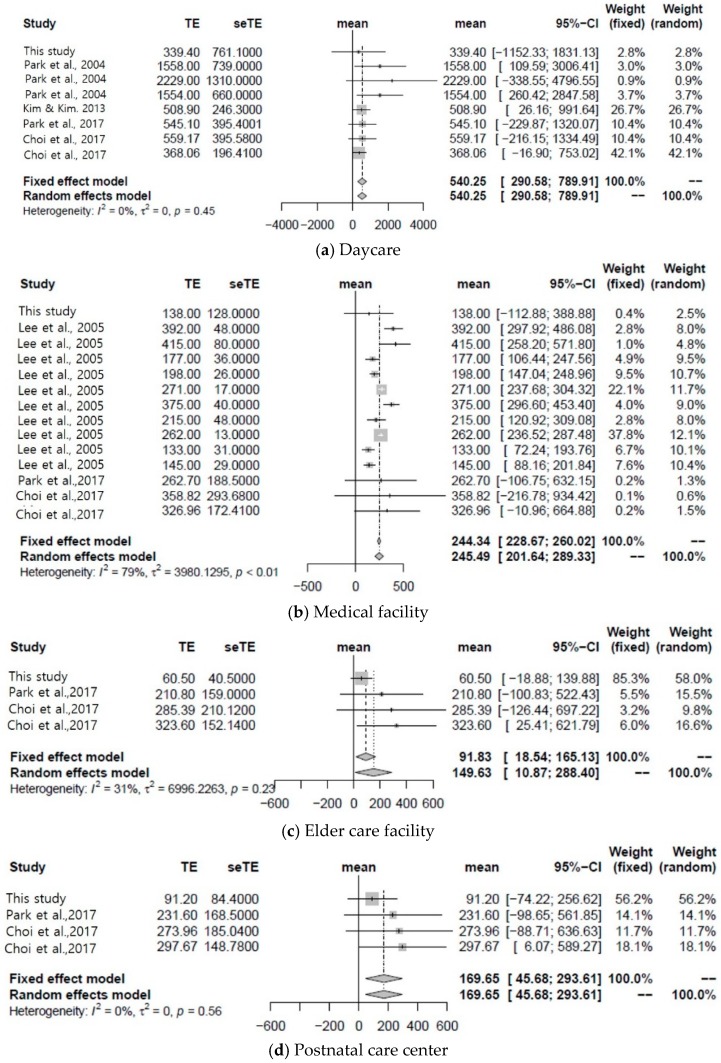
Pooled average concentration of airborne bacteria in each facility (daycare, medical facility, elder care facility, postnatal care center).

Figure 2 shows the results of the combined meta-analysis of the present and previous studies. Values are arithmetic means of indoor airborne bacterial concentration. The mean concentrations of airborne bacteria in the four types of facilities surveyed in this study were lower than those obtained for the facilities in the meta-analysis. The survey was conducted in winter. Comparison with a previous study conducted in summer demonstrated that airborne microbe concentrations in domestic buildings are lower in winter than in summer [21]. The pooled average concentration of airborne bacteria in daycares was 540.25 CFU m^−3^ (95% confidence interval: 290.58–789.91 CFU m^−3^). The pooled average airborne bacterial medical facilities, elder care facilities and postnatal care centers were 245.49 CFU m^−3^ (95% confidence interval: 201.64–289.33 CFU m^−3^), 149.63 CFU m^−3^ (95% confidence interval: 10.87–288.40 CFU m^−3^), and 169.65 CFU m^−3^ (95% confidence interval: 45.68–293.61 CFU m^−3^), respectively.

The high concentration distribution of airborne bacteria in the daycares may be attributed to the comparatively high indoor activity levels there. The relatively high levels of airborne bacteria in the medical facilities can be explained by the high densities of residents (patients) there. In contrast, this is generally not the case with elder care facilities or postnatal care centers.

The airborne bacteria levels in daycares did not exceed the current standard of 800 CFU m^−3^ set by the Ministry of Environment. Therefore, the airborne bacteria levels in many daycares exceed the current limit and the values presented in this study are averages. However, the upper 95% confidence limits for the pooled average concentration closely approached this threshold. Therefore, it is necessary to investigate the airborne bacteria in daycares and establish control measures there. The upper 95% confidence limits of the pooled average airborne bacterial concentrations in each facility were significantly lower than the 800 CFU m^−3^ standard set by the Ministry of Environment. Therefore, unlike daycares, these facilities effectively manage indoor airborne bacterial levels.

### 3.2. Indoor/Outdoor Ratio of Airborne Bacteria

**Figure 3:**
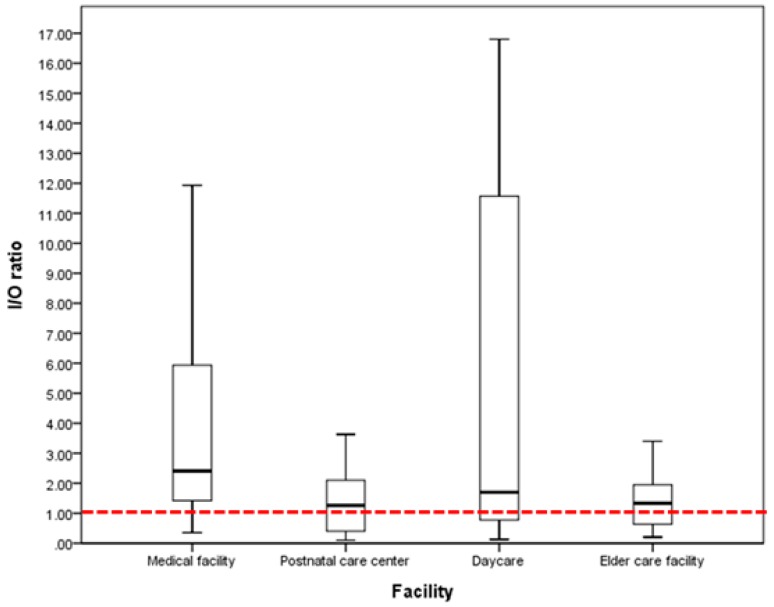
Indoor/outdoor airborne bacterial concentration ratio for each target facility.

Gallup et al. [22] used indoor and outdoor airborne bacterial concentration ratios to determine whether indoor airborne bacteria were mixed with outdoor airborne bacteria. When the concentration ratio was >1, indoor air was contaminated with indoor airborne bacteria. When the concentration ratio was <1, indoor air was contaminated with outdoor airborne bacteria. Indoor airborne bacterial concentrations may be influenced by the external environment including high populations and air pollution from automobile traffic and agricultural activities [13,23]. In this study, the average indoor/outdoor (I/O) ratios of medical facilities, postnatal care centers, daycares, and elder care facilities were 3.7 ± 3.1, 2.0 ± 2.6, 17.1 ± 35.5, and 1.7 ± 1.6, respectively. Therefore, the average indoor/outdoor airborne bacterial concentration ratios were >1 for all facilities. All target facilities had indoor sources of airborne, bacteria which contaminated the indoor air. Figure 3 shows a box plot representing the indoor/outdoor airborne bacterial concentration ratios for each facility. The median value for each facility was >1, which means that >50% of the target facilities had indoor sources of airborne bacteria.

**Figure 4:**
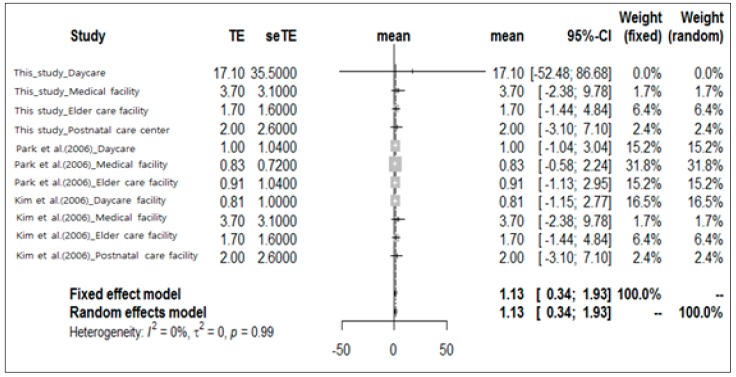
Pooled indoor/outdoor concentration ratios from previous studies.

Only two earlier studies compared indoor and outdoor airborne bacterial concentrations in domestic facilities [24,25]. There are comparatively few studies at the national level on the concentration of outdoor airborne bacteria, because the regulation for airborne bacteria in domestic air has only been stipulated recently in the Indoor Air Quality Control Act set by the Ministry of Environment. This legislation only concerns the concentration of airborne bacteria in the air inside buildings used by pollution-sensitive individuals. Therefore, most of the studies focused only on airborne bacterial concentration in indoor air. The meta-analysis of previous studies considered their limitations and classified target facilities according to their airborne bacterial concentration distributions. It defined all target facilities as those used by pollution-sensitive individuals and calculated the pooled average of indoor/outdoor airborne bacterial concentration ratios in these buildings (Figure 4). The pooled average indoor/outdoor airborne bacterial concentration ratio in the facilities used by pollution-sensitive individuals was 1.13 (95% confidence interval: 0.34–1.93). Average indoor/outdoor airborne bacterial concentration ratios of <1 were found in the medical facilities and elder care facilities surveyed by Park et al. [24] and the daycares surveyed by Kim et al. [25]. Their ratios were 0.83, 0.91, and 0.81, respectively. The average indoor/outdoor concentration ratios for the remaining survey results were >1.0.

Numerous airborne bacteria can be rapidly propagated at optimal temperature, relative humidity, and air flow rate. Therefore, generation patterns may be discontinuous [13,26]. Previous studies have reported that indoor airborne bacteria may be diffused by the residents via indoor cleaning and pet breeding and maintenance. These activities may spread bacteria into the air. Organic household items and food ingredients may serve as nutrient sources for these bacteria. Outdoor bacteria may be introduced into the indoor air flow [15,27,28]. The present study showed that the average indoor/outdoor airborne bacterial concentration ratio was >1. On the other hand, earlier studies reported that outdoor airborne bacteria are a major source of indoor airborne bacteria contamination [15,27,28]. A previous study reported that the most prevalent bacteria is found outdoors [29]. An I/O ration of the total fungal concentration was >1, implying that indoor fungi come from outdoor sources in Southern Taiwan [30]. The indoor bacteria levels were between two and three times higher than the outdoor levels, while the I/O ratio for fungi was >1 in winter and <1 in spring in Southern Poland [31]. The authors concluded that the major sources of these bioaerosols are likely internal, such as building occupants (in this case children and their activities), as well as building materials that host microbiological growth (especially carpets). The authors reported a further study of I/O ratios that showed how air pollutants in the office rooms originate from the indoor air. These results, together with community composition of bacteria, indicate that most of the bacteria present in the studied office building were relatively fresh and of human origin [32]. The most prevalent bacteria found outdoors were gram-positive rods that form endospores. Statistically, the most important meteorological factors related to the viability of airborne bacteria were temperature and UV radiation. However, the aforementioned results suggest that changes in indoor airborne bacterial concentration are enhanced by indoor sources of airborne bacteria in domestic medical facilities, daycares, postnatal care centers, and elder care facilities. Airborne bacterial concentrations can rapidly change in response to various environmental factors. Moreover, the concentration of outdoor airborne bacteria contributes to that of indoor airborne bacteria. Zhu et al. discovered that high correlation coefficients were found between the outdoor relative humidity and outdoor concentration (*p* < 0.01), between indoor and outdoor concentrations (*p* < 0.01), and between indoor relative humidity and indoor concentration (*p* < 0.05), respectively [33].

Outdoor airborne bacterial concentration distributions vary with microbial habitat, measurement time, sampling method, and climatic conditions [34]. In this study, we verified the concentration distribution of outdoor airborne bacteria by using the same sampling methods and the same measurement times. We also determined whether the outdoor airborne bacterial concentration has any influence on indoor airborne bacterial concentration by investigating their relationship according to microbial habitat. The habitats of outdoor airborne bacteria were classified as industrial areas (air pollution), urban areas (high human population densities), and forest areas (pristine) according to the criteria of Fang et al. [23]. Table 2 shows the concentration distribution of indoor and outdoor airborne bacteria in the facilities used by pollution-sensitive individuals. The concentration distribution of outdoor airborne bacteria in urban areas was 74.9 ± 59.7 CFU m^−3^, which was statistically significantly higher than those in the industrial and forested areas (*p* < 0.05). On the contrary, indoor airborne bacterial concentrations did not significantly differ among these areas (*p* > 0.05). There were differences in outdoor but not indoor airborne bacterial concentrations among facilities. According to Kim et al. [35], the outdoor airborne bacterial concentration was significantly higher in green belt areas in spring, summer, and autumn, and in urban areas in autumn. In that study, the concentrations of outdoor airborne bacteria were measured in all four seasons and the target areas were domestic residential, green belt, and roadsides. In winter, there were no statistically significant differences in outdoor airborne bacterial concentration among the three regions. Bacterial proliferation was probably limited during winter because the nutrients accumulated in the soil were depleted by metabolism and assimilation by indigenous bacteria. In the present study, however, outdoor airborne bacterial concentrations were significantly different among the facilities in winter. Nevertheless, the outdoor airborne bacterial concentrations were relatively low at that time. Moreover, the target facilities investigated in this study were used by children, elderly, patients, mothers, and newborns; so, cold air inflow from the exterior was kept to a minimum to keep the interiors of the facilities warm. Therefore, the concentrations of outdoor airborne bacteria did not significantly affect those of indoor airborne bacteria.

Therefore, the concentration of outdoor airborne bacteria must be measured with a view towards collecting data to establish indoor airborne bacteria control plans. Furthermore, the concentration change pattern according to the variations in indoor and outdoor environmental factors must be considered. Since the indoor airborne bacterial concentration may rapidly and substantially change, it is also necessary to evaluate these variations by long-term, continuous measurements.

In view of the indoor/outdoor airborne bacterial concentration ratios and the results of the previous studies, outdoor airborne bacterial concentration must, in fact, influence indoor airborne bacterial concentration. Recently, a literature review by Pearson et al. [36] reported elevated risks of respiratory illnesses with higher bioaerosol exposures in residents near composting sites.

In Korea, where the four seasons are distinct, a study design which factors in seasons may generate comparatively more reliable data and more effectively elucidate the factors affecting indoor airborne bacteria levels. The output of this analysis could be applied towards the development of indoor air quality control plans.

### 3.3. Effect of Indoor Thermal Environment and Environmental Factors

Indoor airborne bacteria are influenced by temperature, relative humidity, ventilation, carbon dioxide levels, oxygen concentration, and anthropogenic dusts [28,37,38,39,40]. In the present study, then, the relationships among the thermal environment (temperature and relative humidity; physical environmental factors), the concentration distribution of airborne bacteria, carbon dioxide concentrations, and dust levels (chemical factors) were examined.

Indoor airborne bacterial concentrations were not related statistically significantly to temperature or relative humidity according to the quartiles for these factors (*p* > 0.05) in daycares (Figure S1). They also were not statistically significantly different from the carbon dioxide concentration, PM_10_, or PM_2.5_, according to the quartiles for these factors (*p* > 0.05) (Figure S1). Indoor airborne bacterial concentrations in the medical facilities (Figure S2) also did not statistically significantly differ with temperature, relative humidity, carbon dioxide concentration, PM_10_, or PM_2.5_ according to the quartiles for these factors (*p* > 0.05). As shown in Figures S3 and S4, in the elder care facilities and postnatal care centers, indoor airborne bacterial concentrations were not correlated with temperature, relative humidity, carbon dioxide concentration, PM_10_, or PM_2.5_ (*p* > 0.05).

These results differ from those reported in previous studies [28,37,38,39,40]. The target facilities of the present study are used by frail individuals who require constant temperature and relative humidity (indoor climate control) regardless of changes in the external environment. In addition, the Indoor Air Quality Control Act of the Ministry of Environment enforces strict regulation of indoor pollutants below a certain level. Therefore, the concentration distributions of each pollutant do not significantly vary among the different types of facility. This result suggests that it is preferable to identify the sources of airborne bacteria and establish a control strategy to reduce the concentrations of airborne bacteria by managing their sources rather than their influencing factors.

## 4. Conclusions

In Korea, the government has set standards and thresholds for the indoor airborne bacteria levels in facilities used by sensitive or high-risk populations (daycares, medical facilities, elder care facilities, and postnatal care centers) to protect fragile individuals against exposure to airborne bacteria. To date, however, no scientific and systematic domestic environmental management plan has been developed or implemented to reduce airborne bacteria. The present study investigated the distribution characteristics and influencing factors of airborne bacteria in these facilities to help establish airborne bacteria reduction measures for them.

This study consisted of a literature review and a field survey on airborne bacteria at target facilities. Airborne bacteria, temperature, relative humidity, fine particulate matter (PM_10_, PM_2.5_), and CO_2_ concentrations were simultaneously measured at each point. To compensate for the lack of representative and reliable data, which could result from a short-term, fragmentary field study, qualitative and quantitative meta-analyses were conducted by linking the literature review results with the concentration measurements.

The pooled average airborne bacterial concentrations at the daycares, medical facilities, elder care facilities, and postnatal care centers were 540.25 CFU m^−3^ (95% confidence interval: 290.58–789.91 CFU m^−3^), 245.49 CFU m^−3^ (95% confidence interval: 201.64–289.33 CFU m^−3^), 149.63 CFU m^−3^ (95% confidence interval: 10.87–288.40 CFU m^−3^), and 169.65 CFU m^−3^ (95% confidence interval: 45.68–293.61 CFU m^−3^), respectively. Daycares clearly presented with the highest indoor airborne bacterial concentrations. The upper 95% confidence limit of the pooled average indoor airborne bacterial concentration at the daycares (789.91 CFU m^−3^) closely approached the 800 CFU m^−3^ threshold set by the Ministry of Environment. Therefore, many daycares may exceed this criterion. The pooled average concentration ratio of indoor to outdoor airborne bacteria was 1.13 (95% confidence interval: 0.34–1.93). Therefore, the sources of indoor airborne bacteria affected the actual indoor airborne bacterial concentrations in daycares, medical facilities, elder care facilities, and postnatal care centers. Unlike previous studies, no significant correlations were found in the present study among the airborne bacterial concentration, indoor physical environmental factors (temperature, relative humidity, and ventilation) and chemical factors (carbon dioxide concentrations and anthropogenic dust levels). The target facilities of the present study are used by fragile individuals, so constant temperature and relative humidity are maintained regardless of changes in the external environment. Moreover, the Indoor Air Quality Control Act of the Ministry of Environment maintains indoor pollutants below a certain level, so the relative differences in indoor pollutant levels among the various facilities are small.

Management of the airborne bacteria levels at daycares should be prioritized. It is preferable to identify the sources of the airborne bacteria by examining each facility. A strategy must be implemented to control airborne bacterial concentrations by managing their sources rather than their influencing factors.

## Figures and Tables

**Table 1:** Airborne bacterial concentration in indoor air in the target facilities.

Facilities	n	Concentration (CFU/m^3^)		F-Value (*p*-Value)	Duncan Coefficient
		Mean	SD *		
Daycares	52	339.4	761.1	4.62 (<0.01)	a
Medical centers	49	138.0	128.0		a,b
Eldercare centers	35	60.5	40.5		b
Postnatal care centers	54	91.2	84.4		b

* SD: Standard deviation.

**Table 2:** Indoor and outdoor airborne bacterial concentrations per region.

Source	Region	n	Concentration (CFU/m^3^)		*p*	Duncan Coefficient
			Mean	SD *		
Indoor	Industrial area	54	118.6	113.5	>0.05	a
	Urban area	64	217.7	594.0		a
	Forested area	72	154.4	372.4		a
Outdoor	Industrial area	54	45.9	35.5	<0.05	a
	Urban area	65	74.9	59.7		b
	Forested area	72	50.1	40.6		a

* SD: Standard deviation.

## Supplementary Materials

The following are available online at https://www.mdpi.com/1660-4601/16/9/1483/s1, Figure S1: Relationship between airborne bacterial concentration and environmental factors in daycares, Figure S2: Relationship between airborne bacterial concentration and environmental factors in medical facilities, Figure S3: Relationship between airborne bacterial concentration and environmental factors in elder care facilities, Figure S4: Relationship between airborne bacterial concentration and environmental factors in postnatal care centers.

## References

[1] Righi E., Aggazzotti G., Fantuzzi G., Ciccarese V., Predieri G. (2002). Air quality and well-being perception in subjects attending university libraries in Modena (Italy). Sci. Total Environ..

[2] Zhang J., Smith K.R. (2003). Indoor air pollution: A global health concern. Br. Med. Bull..

[3] Shinobara N., Kai Y., Mizukoshi A., Fujii M., Kumagai K., Okuizumi Y., Jona M., Yanagisawa Y. (2009). On-site passive flux sampler measurement of emission rates of carbonyls and VOCs form multiple indoor sources. Build Environ..

[4] Missia D.A., Demetriou E., Michael N., Tolis E.I., Bartzis J.G. (2010). Indoor exposure from building materials: A field study. Atmos. Environ..

[5] Owen M.K., Ensor D.S., Sparks L.E. (1992). Airborne particle sizes and sources found in indoor air. Atmos. Environ. A.

[6] Arturo B., Gwen W., Alan M.D., Catherine L.G., Cristina L.H. (2000). School-based identification of asthma in a low-income population. Pediatric Pulmon.

[7] Lacey J., Dutkiewicz J. (1994). Bioaerosols and occupational lung disease. J Aerosol Sci.

[8] Cox C.S., Wathes C.M. (1995). Bioaerosols Handbook.

[9] Macher J.M., First M.W. (1984). Personal air samplers for measuring occupational exposures to biological hazards. Am. Ind. Hygiene Assoc. J..

[10] Chatigny M.A., Macher J.M., Burge H.A., Solomon W.R. (1989). Sampling airborne microorganisms and aeroallergens. Air Sampl. Instrum. Eval. Atmos. Contam..

[11] Law A.K., Chau C.K., Chan G.Y. (2001). Characteristics of bioaerosol profile in office buildings in Hong Kong. Build. Environ..

[12] Golofit-Szymczak M., Gorny R.L. (2010). Bacterial and fungal aerosols in air-conditioned office buildings in Warsaw, Poland—The winter season. Int. J. Occup. Saf. Ergon..

[13] Pastuszka J.S., Paw U.K.T., Lis D.O., Wlazło A., Ulfig K. (2000). Bacterial and fungal aerosol in indoor environment in Upper Silesia. Poland. Atmos. Environ..

[14] Kim Y.S., Roh Y.M., Lee C.M., Kim K.Y., Kim J.C., Jeon H.J., Choi D.M., Kim M.H., Park Y.J. (2007). A study of excess ratio for guideline of indoor air pollutants in classroom of kindergartens. J. Korean Soc. Indoor Environ..

[15] Hyvärinen A., Vahteristo M., Meklin T., Jantunen M., Nevalainen A., Moschandreas D. (2001). Temporal and spatial variation of fungal concentrations in indoor air. Aerosol Sci. Technol..

[16] Ferro A.R., Kopperud R.J., Hildemann L.M. (2004). Source strengths for indoor human activities that resuspend particulate matter. Environ. Sci. Technol..

[17] Koistinen K.J., Edwards R.D., Mathys P., Ruuskanen J., Künzli N., Jantunen M.J. (2004). Sources of fine particulate matter in personal exposures and residential indoor, residential outdoor and workplace microenvironments in the Helsinki phase of the EXPOLIS study. Scand. J. Work Environ. Health.

[18] Kopperud R.J., Ferro A.R., Hildemann L.M. (2004). Outdoor versus indoor contributions to indoor particulate matter (PM) determined by mass balance methods. J. Air Waste Manag. Assoc..

[19] Buttner M.P., Stetzenbach L.D. (1993). Monitoring airborne fungal spores in an experimental indoor environment to evaluate sampling methods and the effects of human activity on air sampling. Appl. Environ. Microbiol..

[20] Choi J.H., Park H.J., Oh Y.J., An J.H., Park J.S., Kim K.R., Sin J.H., Eo S.M., Jung K., Lee J.W., Jang B.K., Son B.S. (2017). Reality analysis and evaluation according to indoor air quality management law of multi-use facilities. J. Odor Indoor Environ..

[21] Moon H.J., An K., Choi M.S. (2012). The status and causes of indoor airborne micro-organisms activities in residential buildings. J. Korean Soc. Living Environ. Syst..

[22] Gallup J.M., Kozak P., Cummins L., Gillman S., Boehm G., Leuschner R.M. (1987). Indoor mold spore exposure: Characteristics of 127 homes in southern California with endogenous mold problems. Advances in Aerobiology.

[23] Fang Z., Ouyang Z., Zheng H., Wang X., Hu L. (2007). Culturable airborne bacteria in outdoor environments in Beijing, China. Microb. Ecol..

[24] Park K.-S., Choi S.-G., Hon J.-K. (2006). The study on the distribution of indoor concentration of microorganism in commercial building. Korean J. Air-Cond. Refrig. Eng..

[25] Kim K.Y., Jang G.Y., Park J.B., Kim C.N., Lee K.J. (2006). Field study of characteristics of airborne bacteria distributed in the regulated public facilities. J. Korean Soc. Occup. Environ. Hyg..

[26] Aylor D.E., Paw U.K.T. (1980). The role of electrostatics in spore liberation by Drechslera turcica. Mycologia.

[27] Lehtonen M., Reponen T., Nevalainen A. (1993). Everyday activities and variation of fungal spore concentrations in indoor air. Int. Biodeterior Biodegrad..

[28] Hargreaves M., Parappukkaran S., Morawska L., Hitchins J., He C., Gilbert D. (2003). A pilot investigation into associations between indoor airborne fungal and non-biological particle concentrations in residential houses in Brisbane, Australia. Sci. Total Environ..

[29] Brągoszewska E., Pastuszka J.S. (2018). Influence of meteorological factors on the level and characteristics of culturable bacteria in the air in Gliwice, Upper Silesia (Poland). Aerobiologia.

[30] Hsu Y.C., Kung P.Y., Wu T.N., Shen Y.H. (2012). Characterization of indoor-air bioaerosols in Southern Taiwan. Aerosol Air Qual. Res..

[31] Brągoszewska E., Mainka A., Pastuszka J. (2016). Bacterial and Fungal Aerosols in Rural Nursery Schools in Southern Poland. Atmosphere.

[32] Brągoszewska E., Biedroń I., Kozielska B., Pastuszka J.S. (2018). Microbiological indoor air quality in an office building in Gliwice, Poland: Analysis of the case study. Air Qual. Atmos. Health.

[33] Zhu H., Phelan P., Duan T., Raupp G., Fernando H.J.S. (2003). Characterizations and relationships between outdoor and indoor bioaerosols in an office building. Chin. Particuol..

[34] Shaffer B.T., Lighthart B. (1997). Survey of culturable airborne bacteria at four diverse locations in Oregon: Urban, rural, forest, and coastal. Microb. Ecol..

[35] Kim K.Y., Kim Y.S., Lee C.M., Cho M.S., Byeon S.H. (2009). Atmospheric distribution characteristics of airborne bacteria in part of Seoul area. J. Korean Soc. Atmos. Environ..

[36] Pearson C., Littlewood E., Douglas P., Robertson S., Gant T.W., Hansell A.L. (2015). Exposures and health outcomes in relation to bioaerosol emissions from composting facilities: A systematic review of occupational and community studies. J. Toxicol. Environ. Health Part B Crit. Rev..

[37] Mancinelli R.L., Shulls W.A. (1978). Airborne bacteria in an urban environment. Appl. Environ. Microbiol..

[38] Hunter C.A., Grant C., Flannigan B., Bravery A.F. (1988). Mould in buildings: The air spora of domestic dwellings. Int. Biodeterior.

[39] Ren P., Jankun T.M., Belanger K., Bracken M.B., Leaderer B.P. (2001). The relation between fungal propagules in indoor air and home characteristics. Allergy.

[40] Balasubramanian R., Nainar P., Rajasekar A. (2012). Airborne bacteria, fungi, and endotoxin levels in residential microenvironments: A case study. Aerobiologia.

