# Supplementary material for: Distribution and Influencing Factors of Airborne Bacteria in Public Facilities Used by Pollution-Sensitive Population: A Meta-Analysis
Source: Int J Environ Res Public Health. 2019 Apr 26;16(9):1483. doi: 10.3390/ijerph16091483 (PMC6539986; doi:10.3390/ijerph16091483)
Supplement: Supplementary file 1 [file ijerph-16-01483-s001.pdf]

International Journal of
Environmental Research and Public Health

Supplemental Figure S1. Relationship between airborne bacterial concentration and environmental factors in daycares.

Supplemental Figure S2. Relationship between airborne bacterial concentration and environmental factors in medical facilities.

Supplemental Figure S3. Relationship between airborne bacterial concentration and environmental factors in elder care facilities.

Supplemental Figure S4. Relationship between airborne bacterial concentration and environmental factors in postnatal care centers.

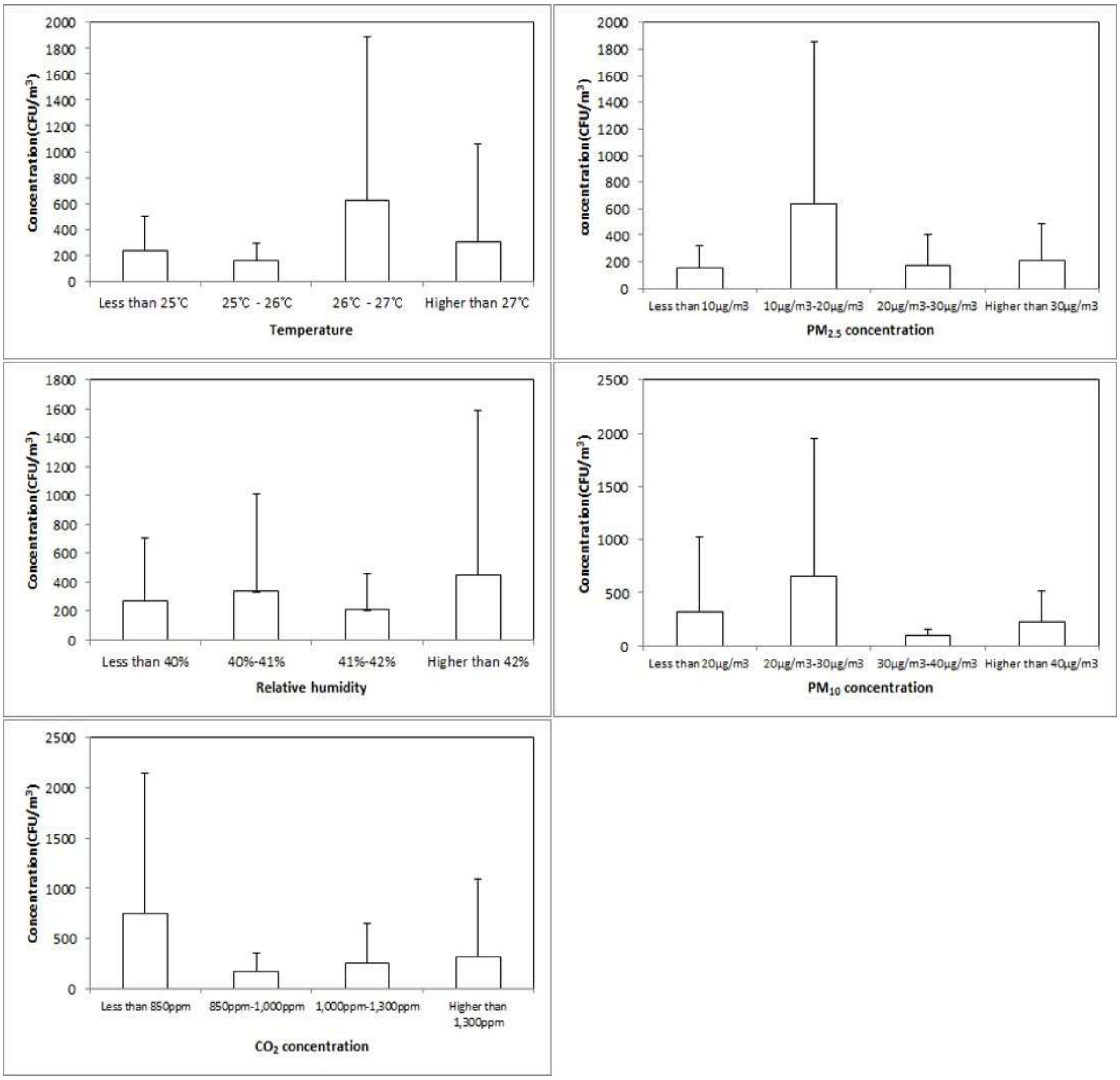

Figure S1. Relationship between airborne bacterial concentration and environmental factors in daycares.

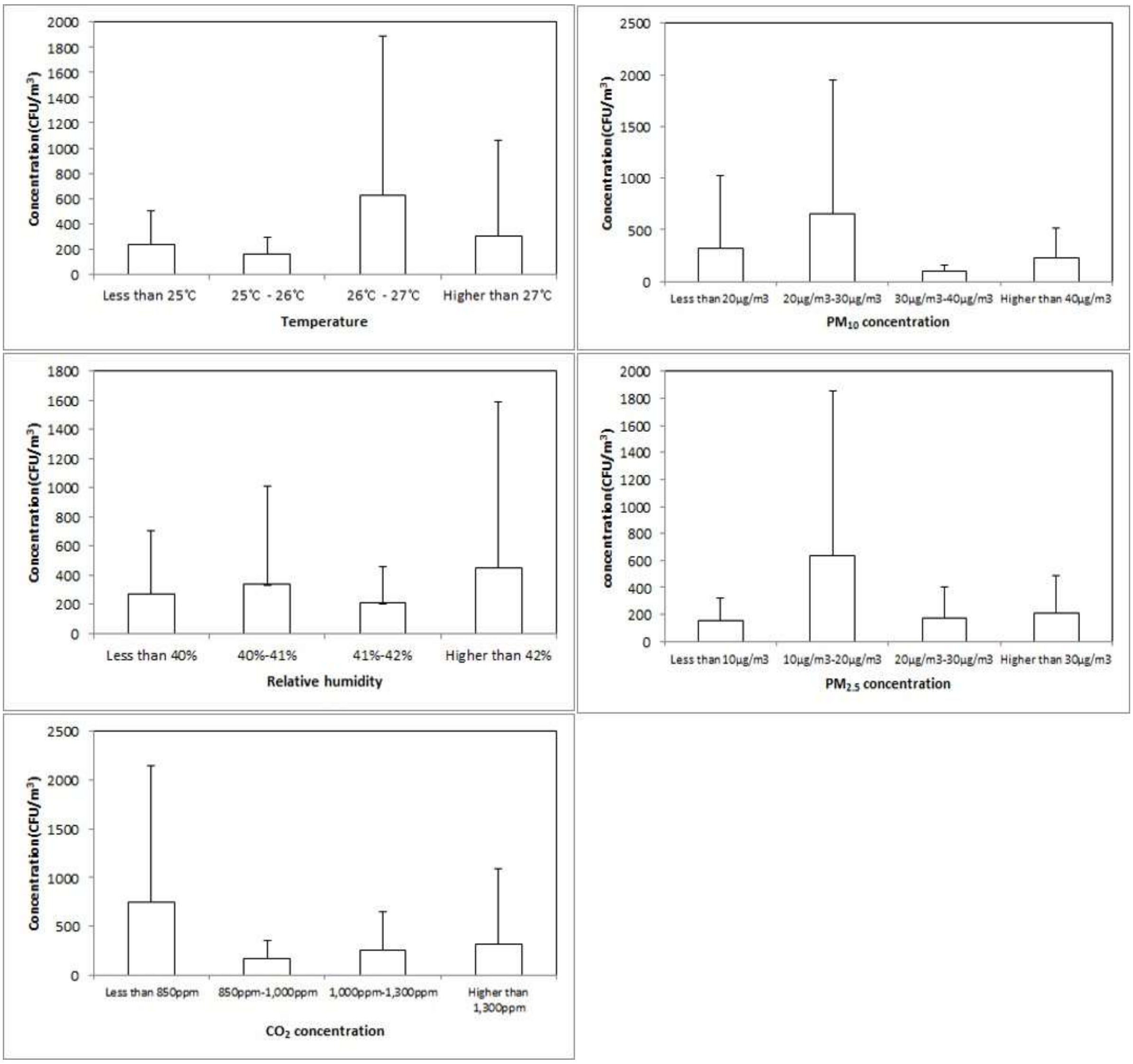

Figure S2. Relationship between airborne bacterial concentration and environmental factors in medical facilities.

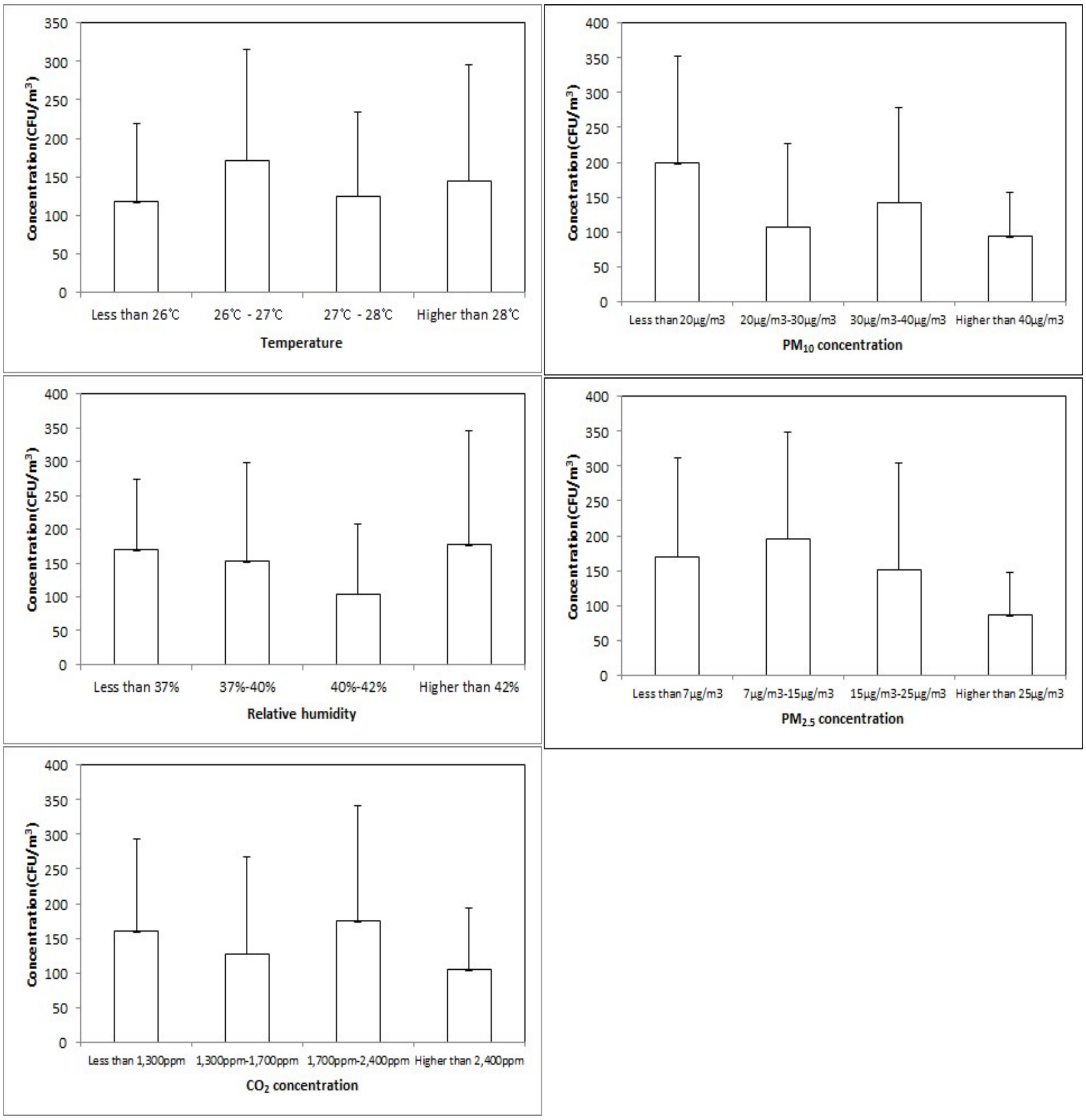

Figure S3. Relationship between airborne bacterial concentration and environmental factors in elder care facilities.

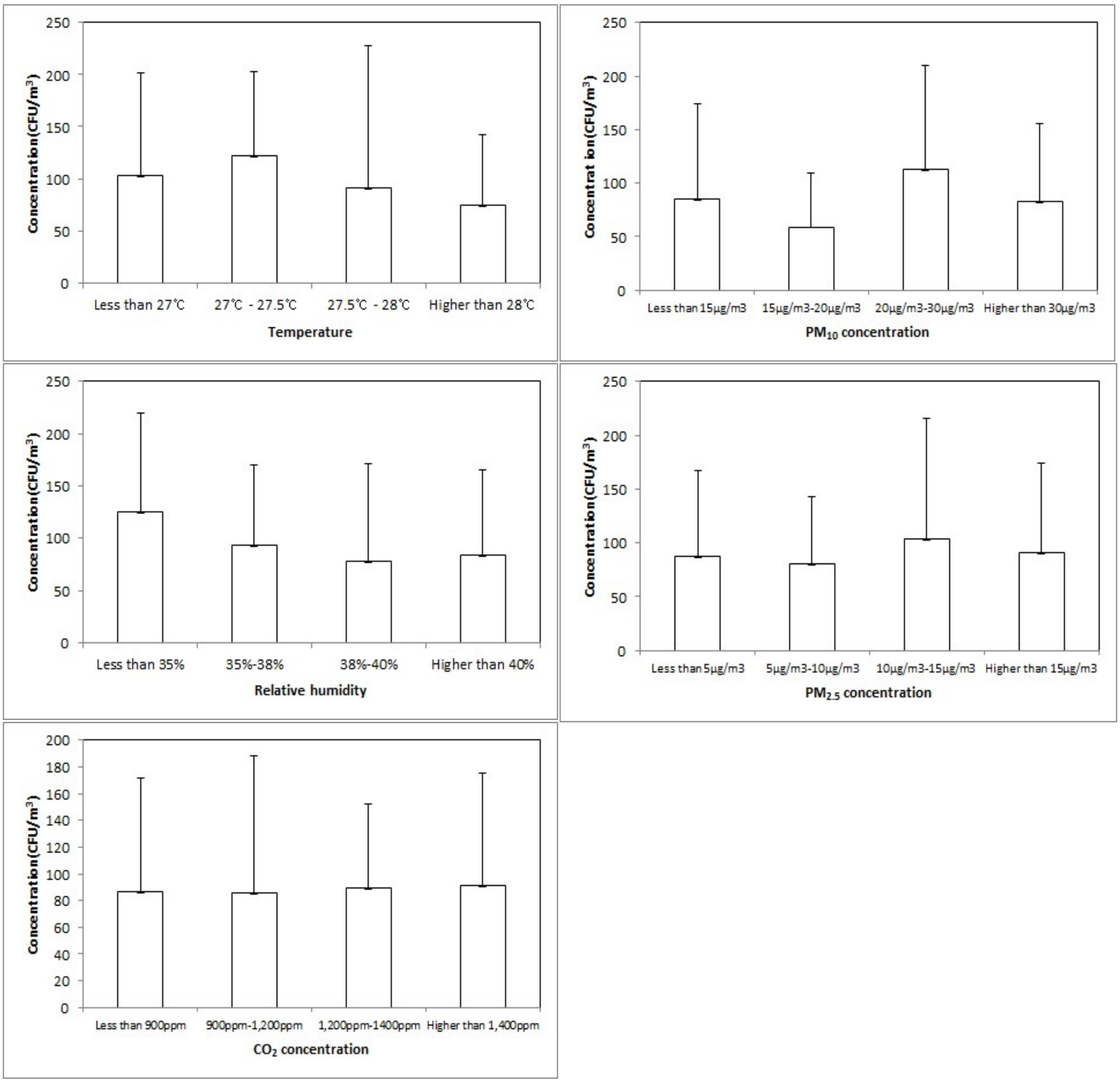

Figure S4. Relationship between airborne bacterial concentration and environmental factors in postnatal care centers.
